# Use of whole-genome sequence data and novel genomic selection strategies to improve selection for age at puberty in tropically-adapted beef heifers

**DOI:** 10.1186/s12711-020-00547-5

**Published:** 2020-05-27

**Authors:** Christie L. Warburton, Bailey N. Engle, Elizabeth M. Ross, Roy Costilla, Stephen S. Moore, Nicholas J. Corbet, Jack M. Allen, Alan R. Laing, Geoffry Fordyce, Russell E. Lyons, Michael R. McGowan, Brian M. Burns, Ben J. Hayes

**Affiliations:** 1 Centre for Animal Science, Queensland Alliance for Agriculture and Food Innovation, University of Queensland, St. Lucia, QLD Australia; 2 School of Health, Medical and Applied Sciences, Central Queensland University, Rockhampton, QLD Australia; 3 Agricultural Business Research Institute, University of New England, Armidale, NSW Australia; 4 Formerly Department of Agriculture and Fisheries, Ayr, QLD Australia; 5 School of Veterinary Science, The University of Queensland, St Lucia, QLD Australia; 6 Neogen, University of Queensland, Gatton, QLD Australia; 7 Formerly Department of Agriculture and Fisheries, Rockhampton, QLD Australia

## Abstract

**Background:**

In tropically-adapted beef heifers, application of genomic prediction for age at puberty has been limited due to low prediction accuracies. Our aim was to investigate novel methods of pre-selecting whole-genome sequence (WGS) variants and alternative analysis methodologies; including genomic best linear unbiased prediction (GBLUP) with multiple genomic relationship matrices (MGRM) and Bayesian (BayesR) analyses, to determine if prediction accuracy for age at puberty can be improved.

**Methods:**

Genotypes and phenotypes were obtained from two research herds. In total, 868 Brahman and 960 Tropical Composite heifers were recorded in the first population and 3695 Brahman, Santa Gertrudis and Droughtmaster heifers were recorded in the second population. Genotypes were imputed to 23 million whole-genome sequence variants. Eight strategies were used to pre-select variants from genome-wide association study (GWAS) results using conditional or joint (COJO) analyses. Pre-selected variants were included in three models, GBLUP with a single genomic relationship matrix (SGRM), GBLUP MGRM and BayesR. Five-way cross-validation was used to test the effect of marker panel density (6 K, 50 K and 800 K), analysis model, and inclusion of pre-selected WGS variants on prediction accuracy.

**Results:**

In all tested scenarios, prediction accuracies for age at puberty were highest in BayesR analyses. The addition of pre-selected WGS variants had little effect on the accuracy of prediction when BayesR was used. The inclusion of WGS variants that were pre-selected using a meta-analysis with COJO analyses by chromosome, fitted in a MGRM model, had the highest prediction accuracies in the GBLUP analyses, regardless of marker density. When the low-density (6 K) panel was used, the prediction accuracy of GBLUP was equal (0.42) to that with the high-density panel when only six additional sequence variants (identified using meta-analysis COJO by chromosome) were included.

**Conclusions:**

While BayesR consistently outperforms other methods in terms of prediction accuracies, reasonable improvements in accuracy can be achieved when using GBLUP and low-density panels with the inclusion of a relatively small number of highly relevant WGS variants.

## Background

The lifetime reproductive capacity of tropically-adapted cows has a major impact on herd productivity and profitability [1–4]. Application of genetic selection for lifetime reproductive capacity has been limited because of its low heritability (0.04 and 0.16 in Tropical Composite and Brahman heifers, respectively) [1] and it is influenced by many environmental and biological factors [4, 5]. Considering associated traits that have a higher heritability and contribute to lifetime reproduction in tropically-adapted beef breeds may be an effective way to select for improved reproductive capacity in cows [1, 6].

Age at puberty is defined as the age at which an animal first ovulates [7, 8]. Age at puberty is moderately heritable (0.52 to 0.57) in tropically-adapted beef heifers [9] and is also favourably genetically correlated (− 0.40 and − 0.33 for Brahmans and Tropical Composites respectively) to lifetime reproductive performance in cows [6], making it ideal for inclusion into selection programs. Moreover, late onset of puberty in beef cattle has a negative impact on a cow’s lifetime reproductive performance and reduces the rate of genetic gain within the herd by directly impacting the generation interval of breeding animals [7]. Selection for reduced age at puberty in cattle can have a favourable genetic impact on female lifetime reproductive performance [1, 10, 11]. A good estimate of age at puberty can be determined by ultrasound scanning the ovaries of heifers at approximately 4 to 6 week intervals to determine age at first corpus luteum (AGECL) [9]. Since AGECL is a difficult and expensive trait to measure, its potential use in commercial herds is limited.

In contrast to AGECL, reproductive maturity score (RMS), a proxy trait for AGECL, is a categorical trait measured on a 0 to 5 scale where 0 = infantile reproductive tract, 1 = small ovarian follicles, 2 = ovarian follicles with a diameter larger than 10 mm, 3 = presence of corpus luteum, 4 = pregnancy to 10 weeks, and 5 = pregnancy longer than 10 weeks [12, 13]. Unlike AGECL, for which multiple measurements are taken, RMS is measured only once, at approximately 600 days of age [12, 13]. Recent studies have shown that RMS is moderately heritable (h^2^ = 0.23) and is highly genetically correlated (r_g_ = − 0.83) to AGECL in tropically-adapted heifers [13], which suggests that it could be used as a proxy for AGECL in genomic evaluations. The advantage of using RMS as a proxy indicator of age at puberty, compared to the highly heritable AGECL trait, is that it is a single measurement that is taken at a similar age as many scanned carcase traits [14]. This reduces the number of times that animals need to be handled, and in turn, reduces associated labour requirements and limits welfare concerns associated with unnecessary handling of stock, both of which can result in significant cost savings in the extensive northern Australian pastoral industry.

Genomic selection can be used to select for difficult and expensive to measure traits, such as age at puberty [15–17]. The accuracy of prediction in genomic selection is related to the number of animals in the reference population for which recorded phenotypes are available [18], the heritability of the trait [19] and relatedness of the populations that have been measured [19, 20]. The beef industry in northern Australia consists primarily of *B. indicus* and *B. indicus *× *B. taurus* crossbred cattle [20, 21]. To accommodate the cross-breeding strategies that are practised in this production system, genomic selection strategies for age at puberty need to be analysed across these breeds and crosses [20]. Recent studies have shown that multi-breed genomic selection is possible in tropically-adapted beef heifers, with no adverse impact on prediction accuracies [20]. However, this study has demonstrated that high-density panels of single nucleotide polymorphisms (SNPs) are required to accurately predict genomic estimated breeding values (GEBV) in these multi-breed populations [20].

Genomic prediction accuracies for single-scan puberty indicators are currently quite low (0.03–0.42) even when high-density SNP panels are used [13, 20]. In order to improve the prediction accuracy of RMS in tropically-adapted beef heifers, improved methodologies of analysis need to be developed. The use of whole-genome sequence (WGS) data has been investigated as a method to improve the accuracy of genomic prediction for some traits [22–24]. However, the use of imputed whole-genome sequence in its entirety does not improve prediction accuracy of genomic selection [23, 24] because the accuracy of estimating millions of SNP effects is limited with the current datasets, and the millions of small errors in the SNP effects compromise accuracy. In addition, the limited improvement of prediction accuracy may be because the currently available high-density panels are sufficient to capture a large proportion of the genetic variance in traits, which may limit the benefit of using whole-genome sequence data in genomic evaluations [23, 24]. However, several recent studies have suggested that the inclusion of pre-selected sequence variants into genomic evaluations may help to improve the prediction accuracy of genomic selection, especially in multi-breed populations [25–27].

In the dairy industry, recent research has investigated the use of novel methods to identify or ‘prune’ WGS variants for inclusion in genomic selection models [25–28]. Raymond et al. [26] showed that the conditional or joint (COJO) WGS pruning methodology successfully improved the prediction accuracy for height in dairy cattle. In contrast, another study reported that COJO WGS pruning reduced prediction accuracy and increased bias in a dairy population, but it was hypothesised that this may be due to the structure of the population used [28]. Multi-genomic relationship matrix (MGRM) analyses have also been used to improve the prediction accuracy of numerically small breeds in and across dairy populations [26]. Compared to a conventional single-genomic relationship matrix (SGRM), the use of a MGRM model where pre-selected SNPs were fit in a separate genomic relationship matrix (GRM), was hypothesised to reduce the effect of bias of individuals from numerically small populations that are incorporated into a GRM populated by a large number of unrelated individuals [26]. Furthermore, in another dairy industry study, non-linear Bayesian analyses were shown to increase the prediction accuracy of genomic evaluations in multi-breed populations [29]. The BayesR models allow SNPs to belong to four different distributions [30], which may help to improve prediction accuracies in multi-breed populations compared to GBLUP models [29]. These three methodologies, use of pre-selected WGS variants, MGRM analyses and BayesR analyses, led to improvements in prediction accuracy in multi-breed populations of dairy cattle [25, 26, 29].

Given the promising results observed for dairy cattle, there may be opportunity to adapt similar methodologies for the north Australian beef industry. Currently, the cost of genotyping large numbers of cattle with high-density genotypes can be considerable and, as such, one of the aims of this research was to investigate if pre-selected whole-genome sequence variants can be incorporated into genomic evaluation models to improve the prediction accuracy of the more cost-effective, low-density panels. We hypothesised that pre-selected WGS variants can be used to improve prediction accuracy for RMS in a multi-breed population of tropically-adapted beef heifers. We tested several WGS pruning techniques in SGRM, MGRM and BayesR models to determine if the accuracy of genomic breeding values for RMS could be improved.

## Methods

### Animals

Fertility records were obtained from two research populations, the Northern Breeding Project research herd from the Beef Cooperative Research Centre for Beef Genetic Technologies (Beef CRC) [9] and the Queensland Smart Futures (SMF) population assembled through the Next Generation Beef Breeding Strategies project [12, 13]. Briefly, 868 Brahman heifers and 960 Tropical Composite heifers with both AGECL phenotype and genotype data were obtained from the Beef CRC. In these herds, AGECL was defined as age (in days) at first corpus luteum, obtained by regular ultrasound scanning of heifers every 4 to 6 weeks. Detailed herd structure, management and data recording are outlined in Johnston et al. [9].

In total, 3695 reproductive maturity scores (RMS) were obtained from the SMF database on heifers from three breeds, Brahman (n = 979), Santa Gertrudis (n = 1802) and Droughtmaster (n = 914). Full information on herd structure, management and data recording is described in Burns et al. [12] and Engle et al. [13].

### Genotypes

Beef CRC heifers were genotyped with the BovineSNP50 BeadChip (Illumina, San Diego, CA) [31] and SMF heifers were genotyped with the 24,121 SNPs from the Geneseek GGP-LD array [20]. Full details on genotype quality control are described in Hayes et al. [20]. Genotypes were imputed up to 728,785 SNPs (Bovine HD array) using the FImpute software [32], and a panel of 1500 individuals from relevant breeds that were genotyped with the Bovine HD array. All genotypes were then imputed to 23 million whole-genome sequence variants using the 1000 Bull Genomes Run6 data base [33] using Eagle [34] for phasing and Minimac3 software [35] for imputation.

Genomic predictions were estimated using three base SNP densities: 6 K (BovineLD array), 50 K (BovineSNP50 BeadChip) and 800 K (BovineHD array). Since animals were genotyped at different SNP densities, the genotypes for the 6 K and 50 K array datasets were constructed by extracting only the SNPs that were present on the commercial BovineLD or BovineSNP50 BeadChip array from the imputed 800 K data.

### Statistical analysis

Three datasets were used in these analyses. The Beef CRC data was split into Brahman and Tropical Composite data, which were used for the discovery of variants associated with AGECL, whereas the SMF heifers were treated as a single population that was used for genomic prediction. The analysis proceeded in two steps:

1. Identification of variants associated with AGECL in Beef CRC populations (Brahman and Tropical Composites) using genome-wide association studies (GWAS) of imputed WGS data in each population separately.
2. Assessment of the accuracy of genomic predictions for RMS in the SMF population when WGS variants pre-selected from the Beef CRC GWAS analyses are added to each analysis.

Genome-wide association studies were performed using the GCTA software [36]. The GWAS model used for the Brahman population was ${\text{AGECL }}\sim {\text{animal}} + {\text{group}} + {\text{dam age}} + {\text{variant}} + {\text{error}}$ and the model used for the Tropical Composites was ${\text{AGECL }}\sim {\text{animal}} + {\text{group}} + B. indicus\;{\text{content}} + {\text{variant}} + {\text{error}}$, where animal is the random animal effect fitted with a GRM, group is contemporary group fitted as a fixed effect, dam age is the age of dam when each test animal was born fitted as a categorical fixed effect, $B. indicus\;{\text{content}}$ is a continuous covariate of *B. indicus* percentage of each Tropical Composite animal, variant is the association between the WGS variant being tested and phenotype and error is the random residual effects of the model. Eight strategies were then applied to preselect WGS variants associated with AGECL in GWAS for inclusion in genomic prediction models (Table 1). All sequence variants were included in pre-selection models and any WGS variant that had a minor allele frequency (MAF) lower than 0.01 were excluded from pre-selection models. Pre-selected WGS variants that were already included on the commercially available SNP panels were excluded from the WGS analysis but continued to be analysed as panel SNPs.

**Table 1:** Description of whole genome sequence (WGS) SNP pre-selection methods

Analysis	Description
CONTROL^a^	Analysis of marker panel only, 6 K, 50 K or 800 K
GWAS SNP pre-selection	
TOP GWAS	All significant variants (*P* ≤ 5.0e−06) from the WGS from each of the beef CRC populations
COJO^b^ GWAS 100	COJO selecting the 100 most significant variants from each of the beef CRC GWAS analyses
COJO^b^ GWAS 250	COJO selecting the 250 most significant variants from each of the beef CRC GWAS analyses
GWAS COJO^b^ CHR	COJO analysis performed within chromosome where significant (*P* ≤ 5.0e−03) variants were selected from each beef CRC GWAS analyses
META SNP pre-selection	
TOP META	All significant (*P* ≤ 5.0e−06) variants from the WGS meta analyses of the beef CRC populations
META COJO^b^ 100	COJO of meta analyses of the beef CRC populations selecting the 100 most significant variants
META COJO^b^ 250	COJO of meta analyses of the beef CRC populations selecting the 250 most significant variants
META COJO^b^ CHR	COJO analysis performed within chromosome where significant (*P* ≤ 5.0e−03) variants were selected from meta-analysis of beef CRC populations

From both single cohort genome wide association study (GWAS) and meta-analysis genome wide association study (META) output

^a^CONTROL = marker panel analysis only, with no WGS variants included in analyses

^b^COJO = conditional or joint analysis in GCTA

Conditional or joint analyses (COJO) were performed in GCTA [37] to pre-select WGS variants using the output from the single cohort GWAS (GWAS) or meta-analysis GWAS output (META). Significance thresholds used to pre-select WGS variants were arbitrarily defined as the most stringent P-value threshold that could be used to identify variants. Initially, a significance threshold of *P* ≤ 5.0e−08 (whole-genome significance) was tested and if no WGS variants met this threshold it was sequentially and arbitrarily lowered (Table 1). For ease of comparison, significance thresholds were equivalent between the TOP GWAS and TOP META analyses and also between the COJO CHR GWAS and COJO CHR META analyses (Table 1).

Single cohort GWAS COJO was performed on each of the Beef CRC datasets and any significant WGS variant identified in either of the datasets was used in genomic predictions. Meta-analyses on the Beef CRC datasets were performed using the program Metal [38]. The P-value threshold model was applied to pre-select WGS variants in the TOP META analyses (see Table 1, for full description). The standard error analysis option was used to perform a meta-analysis in the META COJO analyses. In order to use the standard error analysis in the program Metal, data from each of the META COJO analyses were standardised so that the variant effect ($b$) had a mean of 0 and a standard deviation of 1. This was performed using the following equations:

$$\begin{aligned} & b = \frac{variant\,\,effect}{trait\,\,mean}, \\ & {\text{z}} = - 0.862 + \sqrt {\left( {0.743 - 2.404 \times {\text{log}}\left( {\text{P-value}} \right)} \right)} , \\ & se = \frac{b}{z}. \\ \end{aligned}$$

### Genomic best linear unbiased prediction (GBLUP)

Genomic relationship matrices were constructed for each of the datasets and each marker panel using GCTA [36]. Pre-selected variants were incorporated into each analysis using one of two methods: first, by adding the significant WGS variants and panel SNPs into a single GRM for each analysis (SGRM); and second, by using a multi GRM (MGRM) method where the marker panel GRM remained the same but a second GRM, with only the significant WGS variants, was added and analysed simultaneously. Each GRM was centred using the allele frequencies of the entire population. The GBLUP models for the SGRM (model 1) and MGRM (model 2) are shown below:

1 $${\mathbf{y}} = {\mathbf{Xb}} + {\mathbf{Za}} + {\mathbf{e}},$$

where ${\mathbf{y}}$ is a vector of phenotypes, ${\mathbf{X}}$ is design matrix allocating records to animals, **b** is a vector of fixed effects including the mean, age at measurement fitted as a covariate, contemporary groups fitted as a fixed effect, defined as herd, year and season, ${\mathbf{Z}}$ is an incidence matrix mapping phenotypes to animals, ${\mathbf{a}}$ is a vector of random animal effects and **e** is a vector of random error terms. The distribution of ${\mathbf{a}}$ is assumed to be $N\left( {0, {\mathbf{G}}\upsigma_{\text{g}}^{2} } \right)$, where ${\mathbf{G}}$ is a $n \times n$ matrix of the genomic relationships between individuals estimated using the respective SNPs selected in each analysis [36] and $\upsigma_{\text{g}}^{2}$ is the amount of genetic variance explained by the SNPs in the analysis. Vector ${\mathbf{e}}$ models the random error terms and follows the distribution $N\left( {0, {\mathbf{I}}\upsigma_{\text{e}}^{2} } \right)$, where ${\mathbf{I}}$ is an $n \times n$ identity matrix and $\upsigma_{\text{e}}^{2}$ is the unexplained proportion of variation from the model.

The MGRM model can be written as

2 $${\mathbf{y}} = {\mathbf{Xb}} + {\mathbf{Za}}_{{{\mathbf{Panel}}}} + {\mathbf{Za}}_{{{\mathbf{WGS}}}} + {\mathbf{e}},$$

where parameters and assumptions for the MGRM model (2) are the same as those described for the SGRM model (1), except that ${\mathbf{a}}_{{{\mathbf{Panel}}}}$ is a vector of random animal effects that follows the distribution $N\left( {0, {\mathbf{G}}_{{{\mathbf{Panel}}}}\upsigma_{\text{g}}^{2} } \right)$, where ${\mathbf{G}}_{{{\mathbf{Panel}}}}$ is a $n \times n$ matrix of the genomic relationships between individuals estimated using the panel SNPs and ${\mathbf{a}}_{{{\mathbf{WGS}}}}$ is a vector of random animal effects that follows the distribution $N\left( {0, {\mathbf{G}}_{{{\mathbf{WGS}}}}\upsigma_{\text{g}}^{2} } \right)$, where ${\mathbf{G}}_{{{\mathbf{WGS}}}}$ is a $n \times n$ matrix of the genomic relationships between individuals estimated using the pre-selected WGS variants. The genomic estimated breeding values (GEBV) estimated from the marker panel and the GEBV estimated from the WGS variants were summed to calculate the total GEBV for each animal, which was used to calculate prediction accuracy.

### Bayesian analysis

The BayesR model for each analysis is shown below:

$${\mathbf{y}} = {\mathbf{Xb}} + {\mathbf{Wg}} + {\mathbf{e}},$$

where the covariate terms are the same as described for the GBLUP models and **W** is the standardised genotype matrix (of order equal to the number of phenotypes by number of SNP). Furthermore, ${\mathbf{s}}$ is a vector of SNP effects that follows the distribution ${\mathbf{s}}\sim N\left( {0,\upsigma_{\text{i}}^{2} } \right)$, where $\upsigma_{\text{i}}^{2}$ is one of four distributions: $\upsigma_{\text{i}}^{2}$ = {0, 0.0001, 0.001, or 0.01} $\times\upsigma_{\text{g}}^{2}$, for the ith SNP distribution. The parameter $\upsigma_{\text{g}}^{2}$ is the estimated genetic variance of the trait. Erbe et al. [39] described the two latent parameters that are used in BayesR. The first parameter, ${\text{b}}\left( {{\text{i}}, {\text{k}}} \right)$, defines whether or not the estimated SNP effects follow a normal distribution and ${\text{k}} = \left( {1, 2, 3, 4} \right)$:

$${\text{p}}\left( {{\text{g}}_{\text{i}} | {\text{b}}\left( {{\text{i}},{\text{k}}} \right)} \right) = \left\{ {\begin{array}{*{20}c} {0,} & {{\text{b}}\left( {{\text{i}},1} \right) = 1} \\ {\frac{1}{{\surd 2\uppi \upsigma _{\text{i}}^{2} \left[ {\text{k}} \right]}}\exp \frac{{{\text{g}}_{\text{i}}^{2} }}{{2\upsigma_{\text{i}}^{2} \left[ {\text{k}} \right]}}} & {{\text{b}}\left( {{\text{i}},{\text{k}}} \right) = 1\left( {{\text{k}} = 2,3,4} \right)} \\ \end{array} .} \right.$$

The second parameter, ${\mathbf{Pr}}$ (a vector of length 4) defines the proportion of SNPs that fall into each of the four potential effect groups defined above, and the probability that SNP i falls in each distribution is:

$$\begin{aligned} {\text{p}}\left( {{\text{g}}_{\text{i }} | {\mathbf{Pr}}} \right) & = { \Pr }_{1} \times N\left( {0, 0 \times\upsigma_{\text{g}}^{2} } \right) + {\text{Pr}}_{2} \times {\text{N}}\left( {0, 0.0001 \times\upsigma_{\text{g}}^{2} } \right) \\ & \quad + {\text{Pr}}_{3} \times {\text{N}}\left( {0, 0.001 \times\upsigma_{6}^{2} } \right) + {\text{Pr}}_{4} \times {\text{N}}\left( {0, 0.01 \times\upsigma_{\text{g}}^{2} } \right). \\ \end{aligned}$$

The prior of ${\mathbf{Pr}}$ is sampled from a Dirchlet distribution, ${\mathbf{Pr}}\sim {\mathbf{Dirchlet}}\left( {\varvec{\upalpha}} \right)$, where ${\varvec{\upalpha}}\varvec{ } = \varvec{ }\left[ {1,\varvec{ }1,\varvec{ }1,\varvec{ }1} \right]$. The default option of 50,000 Gibbs chain iterations was used, with the first 20,000 iterations discarded as burn-in, as described by Moser et al. [30]. GEBV were calculated using the following equation described in Hayes et al. [20] (where ${\mathbf{W}}_{{{\mathbf{cands}}}}$ is now the standardised genotype matrix for unphenotyped animals):

$${\text{GEBV}} = {\mathbf{W}}_{{{\mathbf{cands}}}} \widehat{{\mathbf{g}}}.$$

### Prediction accuracy

Five-fold cross-validation was used to determine prediction accuracy. Validation groups were populated by randomly assigning 20% of the SMF animals to one of five validation groups. Individual animals appeared only in a single validation group and these groups were used across all analyses. This random assignment to validation group was designed to reflect the heterogeneous mix of breeds that will most likely occur in genomic evaluations of the northern Australian beef industry.

Correlations between predicted GEBV and RMS phenotypes adjusted for fixed effects were averaged across validation groups for each analysis. Then, average correlations were divided by the square root of the heritability of RMS to calculate the prediction accuracy. The heritability estimates used in the calculation of accuracies were obtained from the 800 K CONTROL analysis (Table 2). Control analyses for each of the methods used a model that included only information from the marker panel and no added WGS variants. The variance components for each scenario are averaged over each of the five reference populations and the standard errors presented are the standard errors of the mean of the average estimates. Prediction bias was calculated as the regression coefficient between adjusted phenotype (y-axis) and GEBV (x-axis) in each of the analyses.

**Table 2:** Variance components for Smart Futures heifers for reproductive maturity score (RMS) estimated on the 800 K marker panel with standard errors

GRM	Analysis	Vp (se)	Va (se)	Ve (se)	h^2^ (se)	WGS Va (se)	WGS h^2^ (se)	Total h^2^ (se)
SGRM	CONTROL	1.11 (0.01)	0.22 (0.01)	0.89 (0.01)	0.20 (0.01)	–	–	–
	TOP GWAS	1.11 (0.01)	0.22 (0.01)	0.89 (0.01)	0.20 (0.01)	–	–	–
	TOP META	1.11 (0.01)	0.22 (0.01)	0.89 (0.01)	0.20 (0.01)	–	–	–
	COJO GWAS 100	1.11 (0.01)	0.22 (0.01)	0.89 (0.01)	0.20 (0.01)	–	–	–
	COJO GWAS 250	1.11 (0.01)	0.22 (0.01)	0.89 (0.01)	0.20 (0.01)	–	–	–
	GWAS COJO CHR	1.11 (0.01)	0.22 (0.01)	0.89 (0.01)	0.20 (0.01)	–	–	–
	COJO META 100	1.11 (0.01)	0.22 (0.01)	0.89 (0.01)	0.20 (0.01)	–	–	–
	COJO META 250	1.11 (0.01)	0.22 (0.01)	0.89 (0.01)	0.20 (0.01)	–	–	–
	META COJO CHR	1.11 (0.01)	0.22 (0.01)	0.89 (0.01)	0.20 (0.01)	–	–	–
MGRM	TOP GWAS	1.11 (0.00)	0.20 (0.01)	0.90 (0.02)	0.18 (0.01)	0.012 (0.002)	0.012 (0.002)	0.19 (0.011)
	TOP META	1.13 (0.01)	0.19 (0.01)	0.90 (0.01)	0.17 (0.01)	0.042 (0.007)	0.036 (0.008)	0.21 (0.012)
	COJO GWAS 100	1.11 (0.01)	0.20 (0.01)	0.89 (0.01)	0.18 (0.01)	0.010 (0.000)	0.012 (0.002)	0.20 (0.010)
	COJO GWAS 250	1.11 (0.01)	0.19 (0.01)	0.89 (0.01)	0.17 (0.01)	0.028 (0.007)	0.024 (0.005)	0.20 (0.010)
	GWAS COJO CHR	1.11 (0.01)	0.20 (0.01)	0.89 (0.01)	0.18 (0.01)	0.014 (0.007)	0.010 (0.006)	0.20 (0.010)
	COJO META 100	1.11 (0.01)	0.20 (0.01)	0.89 (0.01)	0.18 (0.01)	0.022 (0.002)	0.018 (0.002)	0.20 (0.012)
	COJO META 250	1.11 (0.01)	0.18 (0.01)	0.89 (0.01)	0.16 (0.01)	0.034 (0.002)	0.032 (0.002)	0.20 (0.011)
	META COJO CHR	1.10 (0.00)	0.20 (0.01)	0.90 (0.01)	0.18 (0.01)	0.016 (0.002)	0.012 (0.002)	0.19 (0.010)
BAYES	CONTROL	1.09 (0.00)	0.19 (0.01)	0.90 (0.01)	0.17 (0.01)	–	–	–
	TOP GWAS	1.09 (0.00)	0.19 (0.01)	0.90 (0.01)	0.17 (0.01)	–	–	–
	TOP META	1.09 (0.00)	0.19 (0.01)	0.90 (0.01)	0.17 (0.01)	–	–	–
	COJO GWAS 100	1.09 (0.00)	0.19 (0.01)	0.90 (0.01)	0.17 (0.01)	–	–	–
	COJO GWAS 250	1.09 (0.00)	0.19 (0.01)	0.90 (0.01)	0.17 (0.01)	–	–	–
	GWAS COJO CHR	1.09 (0.00)	0.19 (0.01)	0.90 (0.01)	0.17 (0.01)	–	–	–
	COJO META 100	1.09 (0.00)	0.19 (0.01)	0.90 (0.01)	0.17 (0.01)	–	–	–
	COJO META 250	1.09 (0.00)	0.18 (0.01)	0.90 (0.01)	0.17 (0.01)	–	–	–
	META COJO CHR	1.09 (0.00)	0.18 (0.01)	0.90 (0.01)	0.17 (0.01)	–	–	–

Estimated phenotypic variance (Vp), additive variance (Va), environmental variance (Ve) and heritability (h^2^) estimated for single GRM (SGRM), Bayes R (BAYES) analyses and multi-GRM (MGRM) analyses. Whole-genome sequence (WGS) variants additive variance (WGS Va), WGS variants heritability (WGS h^2^) and total heritability (total h^2^) estimated in the multiple genomic relationship matrix (MGRM) analyses in the Smart Futures heifers 800 K marker panel (standard errors (se) in parentheses)

Model significance was tested using a linear model function in R [40] that tested panel density, GWAS type (GWAS or META), WGS variant pre-selection method and type of analysis, i.e. SGRM, MGRM and BayesR, to determine the effect of these factors on prediction accuracy for RMS. Pair-wise comparisons between factor levels were estimated using the emmeans package in R [41]. When performing significance testing, the CONTROL groups were removed from the analysis because CONTROL was confounded in the MGRM analysis since the MGRM CONTROL was equivalent to the SGRM CONTROL.

## Results

Initial statistical investigations showed that RMS is an approximately normally distributed categorical trait with a mean of 2.27 and a range from 0 to 5, which indicates that variation in this trait exits between the Smart Futures heifers. Variance component estimates were similar between marker panels, therefore for ease of reference, only the results estimated from the 800 K panel are presented in Table 2. The addition of WGS variants had little effect on the heritability estimates, across all marker panels.

**Fig. 1:**
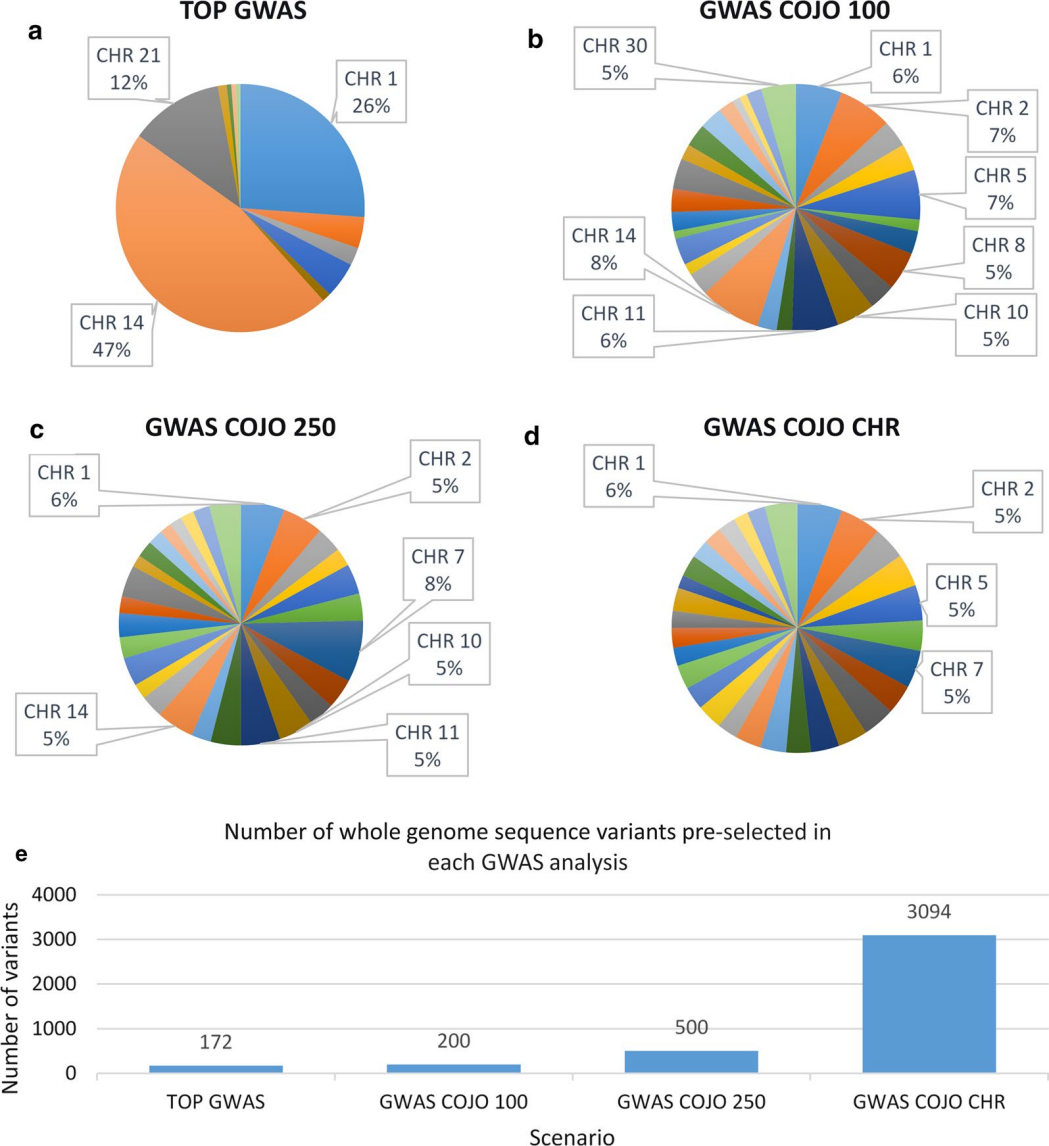
Number of pre-selected whole-genome SNPs from single cohort genome-wide association studies. Numbers of pre-selected whole-genome sequence (WGS) SNPs in each of the single cohort genome-wide association studies (GWAS) with the 6 K panel by chromosome (CHR) (**a**–**d**) and the total number of preselected WGS SNPs in each analysis (**e**)

**Fig. 2:**
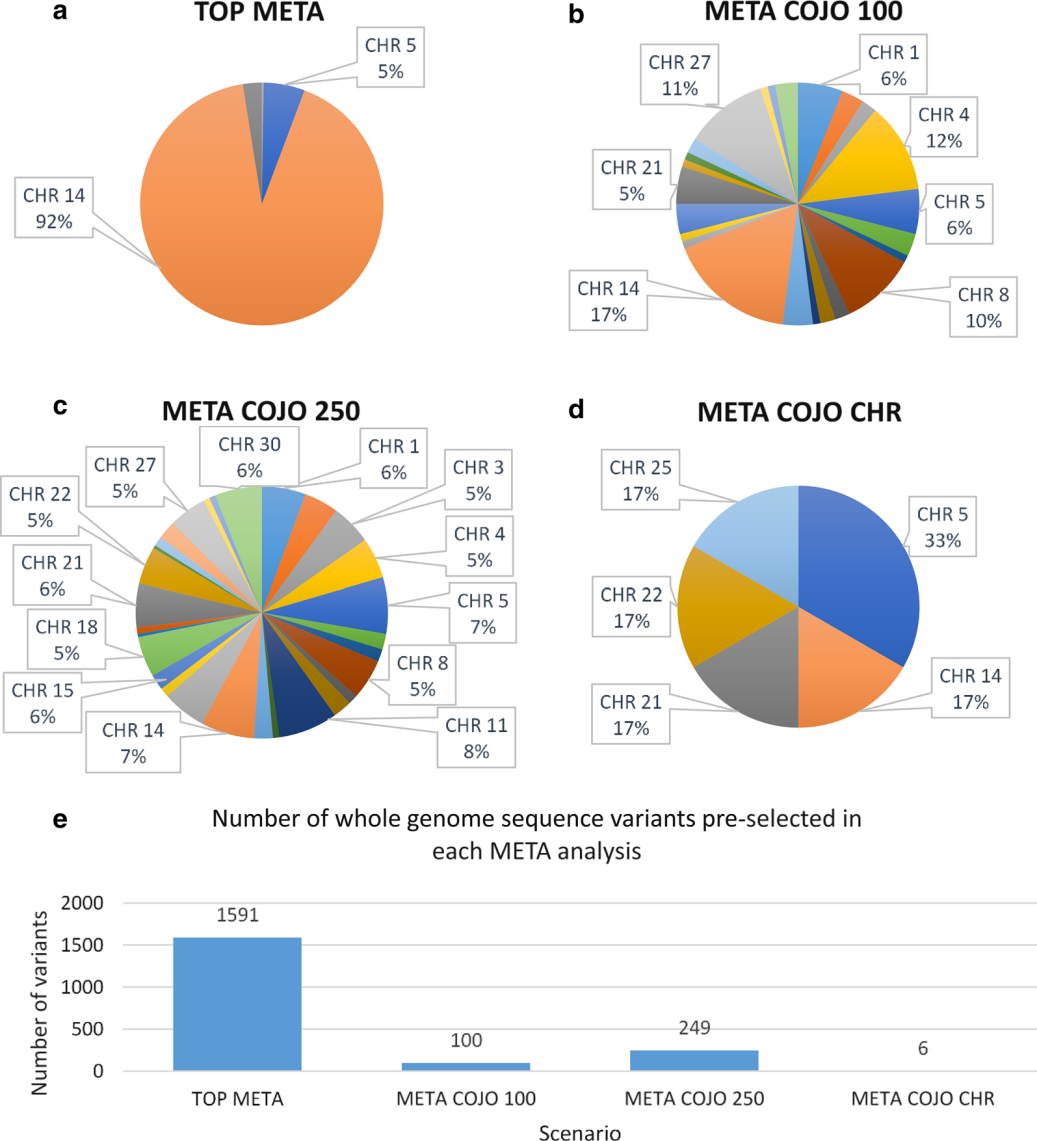
Number of pre-selected whole-genome SNPs from meta-analysis genome-wide association studies. Numbers of pre-selected whole genome sequence (WGS) SNPs in each of the meta-analysis genome-wide association studies (META) with the 6 K panel by chromosome (CHR) (**a**–**d**) and the total number of preselected WGS SNPs in each analysis (**e**)

The numbers of variants selected from each chromosome were similar across the three panels (Figs. 1 and 2; the results from the 6 K panel are displayed). Compared to the GWAS results (Fig. 1), the META analyses generally resulted in fewer WGS variants being selected from each chromosome, with the exception of the TOP META analysis (Fig. 2). The TOP META analysis identified the largest number of pre-selected WGS variants among all META analyses, however, all WGS variants were on four chromosomes only, 3, 5, 14 and 21. The majority of the 1591 WGS variants pre-selected in the TOP META analysis were located on chromosome 14 (92%). Similar to the GWAS results (Fig. 1), COJO analysis of the META results resulted in a reduced number of variants selected from each chromosome. Unlike the COJO GWAS results, the META COJO CHR analysis identified fewer significant variants than the other META pruning methods, even with a less stringent P-value threshold (*P* ≤ 5.0e−03). Of the six variants identified in this pre-selection method, two were located on chromosome 5, and one on each chromosome 14, 21, 22 and 25 (Table 3).

**Table 3:** Annotation of significant pre-selected whole-genome sequence variants

Chromosome	Position	Annotation	Gene	SNP
5	46074974	Intergenic		rs719636104
5	70786467	Intergenic		s133829475
14	25315124	Intergenic	*PLAG1*	rs133340360^a^
21	6816909	Intron	*ADAMTS17*	rs109115540
22	25147635	Intron	*CNTN6*	rs382635721
25	1561873	Intron	*ZNF598*	rs516692605

Annotation of pre-selected variants identified in the 6 K meta conditional or joint analysis (COJO) by chromosome

^a^305 kb from gene

**Fig. 3:**
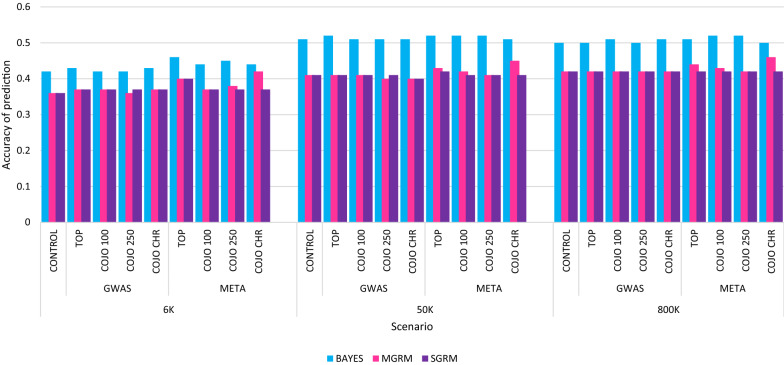
Prediction accuracy for reproductive maturity score (RMS) in the Smart Futures (SMF) heifers. Prediction accuracy for various whole-genome sequence (WGS) SNP pre-selection strategies and marker panel density for BayesR and genomic best linear unbiased prediction (GBLUP) single genomic relationship matrix (SGRM) and GBLUP multiple genomic relationship matrix (MGRM) analyses. CONTROL estimates are the prediction accuracies estimated with no WGS SNP included in the models. MGRM CONTROL analyses are the SGRM CONTROL estimates

The prediction accuracies of all the BayesR analyses, including the CONTROL analyses, were higher than for any of the within-panel GBLUP analyses (Fig. 3). The greatest improvement in prediction accuracy using BayesR was obtained with the higher density panels, as also observed in the GBLUP results. Unlike the GBLUP results, the 50 K panel showed slightly better prediction accuracies than the 800 K analysis when using BayesR. Estimates of the CONTROL analyses in the 50 K and 800 K BayesR analyses were 0.51 and 0.50, respectively. Inclusion of WGS variants in the 50 K and 800 K analyses did not improve prediction accuracy in either BayesR or GBLUP. Prediction accuracy in the 6 K BayesR CONTROL analysis was 0.42, which is similar to the 800 K GBLUP CONTROL or the 6 K COJO CHR meta-analysis GBLUP analyses. Similar to the GBLUP results, the 6 K panel benefitted most from the inclusion of WGS variants in BayesR analyses.

Estimates of prediction bias were not significantly affected by the density of the panels or the WGS variant pre-selection strategy used. Type of analysis, GBLUP or BayesR, influenced the estimates of bias in this study, however these differences were not statistically significant. Estimates of bias regression coefficients for the GBLUP analysis ranged from 0.84 to 0.94 and there were no significant differences between each of the GBLUP analyses. Estimates of bias regression coefficients in BayesR analyses ranged from 1.05 to 1.23 and, again, there were no significant differences between each of the BayesR analyses.

When examining the effect of each potential factor in the model on prediction accuracy; validation group, the type of GWAS, analysis and marker panel all had a significant (*P *< 0.001) effect. The effect of the WGS variant pre-selection method was bordering upon statistical significance (*P *< 0.06) in these analyses. Furthermore, the interactions between analysis and marker panel (*P* < 0.001) and analysis and type of GWAS (*P* < 0.01) had significant effects on the prediction accuracy for RMS in these models. Pairwise comparisons of factor levels showed that the prediction accuracy of RMS was significantly different (*P* < 0.001) between the GBLUP (SGRM and MGRM) analyses and the BayesR analyses, but there was no difference between the SGRM and MGRM GBLUP analyses. Panel density also had a significant effect on prediction accuracy in these models, and the pairwise comparisons showed that there was a significant difference between the 6 and 50 K and the 6 K and 800 K panels (*P* < 0.001), but no difference between the 50 and 800 K panels. There was also a significant difference in accuracy between the GWAS and META WGS variant pre-selection methods (*P* < 0.001).

## Discussion

The hypothesis underlying this study was that pre-selected WGS variants can be used to improve prediction accuracy of RMS in a multi-breed population of tropically-adapted beef heifers. Our results show that, in some cases, pre-selection of WGS variants and novel analysis methodologies were able to improve the accuracy of prediction of RMS without increasing prediction bias. The greatest improvement in prediction accuracy for RMS in the SMF heifers came from using the higher density panels, 50 K and 800 K, irrespective of the inclusion of WGS variants. The use of higher density panels significantly improved prediction accuracy compared to the 6 K panel, however, we found no significant difference between the 50 and 800 K prediction accuracies. Similarly, Raymond et al. [25] have shown that inclusion of WGS variants had little effect on prediction accuracy of the 50 K or 800 K panels in dairy cattle. The range of linkage disequilibrium (LD) in beef cattle is relatively long and the 50 K panel may be sufficient to capture this variation [39], which would explain the lack of significant improvement in prediction accuracy with increased marker density. However, the extent of LD between breeds depends largely on the effective population size of each breed [20]. In the north Australian pastoral industry, many breeds are produced commercially, including large numbers of *B. indicus* and *B. taurus* crossbreeds [21]. The genetic divergence between *B. indicus* and *B. taurus* breeds is potentially large and more research is required to determine the most appropriate density of panels in this situation.

Increasing the size of the reference set (animals genotyped and phenotyped) has been shown to improve the accuracy of GEBV, including in tropical beef breeds [20, 42]. In populations with many breeds, accumulating large reference populations for each breed may be challenging. An alternative is to run a multi-breed analysis. Recently, Hayes et al. [20] estimated within- and multi-breed prediction accuracy of another puberty proxy trait, the corpus luteum score, in the same cohort of Smart Futures heifers as those used in our study. Prediction accuracies were calculated using both a within-breed and multi-breed reference population and the results demonstrated that prediction accuracy estimates were improved for each breed using a multi-breed reference population rather than a within-breed reference population [20]. In the extensive northern Australian beef industry, accurate recording of the breed composition of individual animals can be limited, which may adversely affect the prediction accuracy of genomic evaluations. Our aim was to determine if accurate multi-breed GEBV can be predicted to aid the development of genomic evaluation programs in these industries. Consequently, our study used both multi-breed reference and validation populations. The method that we used to randomly assign reference and validation populations did not consider breed, and it is possible that breed composition varied within validation group. We found that validation group had a significant effect on the prediction accuracy of RMS and some of this variation may be due to breed effects. In the future, it may be necessary to consider methods that more accurately account for breed effects in multi-breed genomic evaluations.

Bayesian methodologies resulted in the highest prediction accuracies for RMS, regardless of the panel density and WGS variant pre-selection strategy. In our study, Bayesian analyses had significantly higher prediction accuracies across all markers and pre-selection methods than the GBLUP analyses. A number of other studies have shown that genomic selection using Bayesian methods performed better in across-breed predictions than GBLUP analyses [29, 39], which could be due to differences in the model assumptions between the two methods [29]. One of the model assumptions of GBLUP analyses is that all SNPs within the GRM have a small effect on the trait of interest [29] and, as such, GBLUP uses all the SNPs equally to make genomic predictions [29]. When estimating within-breed genomic predictions, GBLUP performs equivalently to Bayesian methods, due to long-range LD that usually exists within breeds [29]. However, in multi-breed analyses recombination can affect the associations between SNPs and causative mutations, breaking up the long-range LD, and reducing the prediction accuracy of GBLUP analyses [29]. Model assumptions of the Bayesian analyses allow SNPs to have a zero effect within the analysis, which means that each analysis is being performed by using only SNPs that are in LD with SNPs that have an effect on RMS [29, 39]. Thus, by using the information from SNPs that only affect the trait, Bayesian models have an improved ability to estimate genomic differences between animals of genetically diverse breeds [29, 39].

The use of a MGRM model in some of the GBLUP analyses showed potential for improving prediction accuracy for RMS in the SMF heifers. A MGRM model for genomic analyses can improve the accuracy of GBLUP predictions in dairy populations [26, 27]. The use of a second GRM that consists of pre-selected variants, allows these variants to have a larger variance than they would have if they were fitted in a larger SGRM [27]. In a large SGRM, the effect of pre-selected WGS variants will be regressed more towards the mean than if they were fitted in a MGRM model. Since these variants have been pre-selected from WGS because of their significant association with RMS, the SGRM may be biasing the effect of these pre-selected WGS variants. This may affect the ability of the algorithm to accurately estimate SNP effects in SGRM models, reducing the potential to further increase accuracy.

Prediction accuracies obtained with meta-analysis GWAS were slightly higher than with single cohort GWAS analyses, particularly in the GBLUP MGRM models. Meta-analyses are used to account for differences in population size and trait measurements when combining datasets from unrelated populations [43]. Linkage disequilibrium in multi-breed populations is expected to be shorter, which can improve the precision with which SNPs can be identified in GWAS [43]. Thus, by combining the Beef CRC populations in a meta-analysis, we may have improved the ability to identify WGS variants that affect AGECL, resulting in increased prediction accuracies in the META analyses. Furthermore, in this study, GWAS variant pre-selection techniques considered neither the differences in the variance of AGECL between the Beef CRC datasets, nor the differing numbers of animals used for GWAS analysis within each of the Beef CRC datasets. The slight improvement in META analysis prediction accuracies in our study, compared to single cohort GWAS pre-selection, suggests that when pre-selecting variants from combined datasets of unrelated animals, it may be beneficial to use meta-analyses. Teissier et al. [43] provided support for this hypothesis by showing that meta-analysis of GWAS results were beneficial in identifying variants in whole-genome sequence data, and resulted in the most accurate multi-breed genomic evaluation.

Of the eight WGS variant pre-selection strategies, two of the meta-analysis pre-selection strategies that we used resulted in improved prediction accuracies in the GBLUP analyses, TOP and COJO CHR. Figure 2 shows that 1591 and 6 SNPs were selected from these analyses, respectively. Furthermore, both of these pre-selection strategies selected variants from very few chromosomes, i.e. BTA14 (92%), BTA5 (5%) and BTA21 (3%) for TOP and BTA5 (33%), BTA14 (16.75%), BTA21 (16.75%), BTA22 (16.75%) and BTA25 (16.75%) for COJO CHR, respectively. The arbitrary cut-off values set in the COJO 100 and COJO 250 analyses may have resulted in a number of less significant WGS variants being pre-selected from a large number of chromosomes. In GBLUP analyses, all SNPs are assumed to influence the trait [29] and the inclusion of SNPs that do not have a significant effect on the trait will potentially reduce prediction accuracy. In contrast, the pre-selection of variants from a meta-analysis using significance value thresholds (e.g. TOP and COJO CHR) only selected variants with a significant association with the RMS trait, which increased prediction accuracies in the GBLUP analyses.

The most significant improvement in prediction accuracy due to the inclusion of WGS variants from the meta-analysis was observed when the lower density panels, particularly the 6 K MGRM models, were used. Of these models, the COJO CHR pre-selection had the highest prediction accuracy in the GBLUP analyses from the inclusion of only six WGS variants. Three of these variants were identified on introns of the *ADAMTS17*, *CNTN6* and *ZNF598* genes. An association between *ADAMTS17* and height has been reported in horses [44], dairy cattle [45] and humans [46]. Increased hip height has been identified as adversely genetically correlated with puberty in beef cattle [47], which may explain the significant effect of *ADAMTS17* on AGECL in this study. Furthermore, a recent study has identified a SNP in *ADAMTS17* that is significantly associated with age at puberty in a population of tropically-adapted heifers [48]. Similarly, *ZNF598* is associated with low fertility in dairy cattle [49]. The variant in *CNTN6* is associated with neural development and spatial learning in mice and is differentially expressed between the sexes in the developing brain [50], and to the authors’ knowledge, it has not been associated with fertility in cattle. In addition to the variants in these three introns, one intergenic variant was identified on BTA14 that was within a 305-kb region of the *PLAG1* gene. Due to its proximity, this variant is very likely tracking a mutation in the *PLAG1* gene or its regulators. Recent research has shown that *PLAG1* is associated with age at puberty in tropically-adapted cattle [51]. The inclusion of these six SNPs from the meta-analysis COJO CHR multi-GRM model, increased the prediction accuracy of the GBLUP 6 K analysis to 0.42, which is the same as the estimated prediction accuracy of the GBLUP 800 K control. The economic effect of using a lower density panel for genotyping while achieving the same accuracy as with higher density panels may have wide-reaching implications for applied genomic predictions, and as such, this finding warrants further investigation.

While the results from this study suggest that the use of pre-selected WGS variants and novel analysis methodologies can be used to improve the prediction accuracy for RMS in a multi-breed population of tropically-adapted heifers, it is worth noting that these are not true across-breed GEBV. Across-breed GEBV can only be calculated with direct breed comparisons through mixed breed cohorts, which were not available in the SMF dataset. As each of the three breeds of heifers from the Smart Futures dataset were managed separately, in our analyses the definition of the contemporary group accounted for breed effect. The development of an across-breed GEBV for the northern Australian beef industry will require the collection of genotypes and phenotypes on animals that are produced in multi-breed cohorts, in which breed performance can be directly compared. When these datasets become available, further research will be necessary to develop methods to predict across-breed GEBV and methods to account for differences between breeds within these analyses.

## Conclusions

Both BayesR and some GBLUP MGRM analysis with pre-selected WGS variants resulted in improved prediction accuracies for RMS in this population of heifers. Generally, BayesR analyses had higher prediction accuracies than GBLUP, and the addition of WGS variants had very little effect on BayesR estimates. Pre-selection of WGS variants was most beneficial in the GBLUP analyses, particularly when using variants that were pre-selected by using meta-analysis COJO CHR in multi-GRM analyses. The most pronounced improvements in prediction accuracy were observed when genotypes were based on the 6 K panel for both the BayesR and GBLUP methods. The 6 K panel is the most cost-effective option and, if the prediction accuracy of this panel can be improved, there will be a financial benefit to end-users. More RMS phenotypes will be required to improve accurate detection of WGS variants that can explain variation in RMS across a number of tropically-adapted breeds and the best methods to use this data in genomic prediction analyses.

## Data Availability

The datasets used and/or analysed during the current study are available from the corresponding author on reasonable request. GWAS summary statistics are also available upon request.
